# gamUnet: designing global attention-based CNN architectures for enhanced oral cancer detection and segmentation

**DOI:** 10.3389/fmed.2025.1582439

**Published:** 2025-07-23

**Authors:** Jinyang Zhang, Hongxin Ding, Runchuan Zhu, Weibin Liao, Junfeng Zhao, Min Gao, Xiaoyun Zhang

**Affiliations:** ^1^School of Computer Science, Peking University, Beijing, China; ^2^Key Laboratory of High Confidence Software Technologies, Ministry of Education, Peking University, Beijing, China; ^3^School and Hospital of Stomatology, Peking University, Beijing, China

**Keywords:** oral squamous cell carcinoma (OSCC), segmentation, image processing, image classification, convolutional neural networks, deep learning–artificial intelligence, artificial intelligence

## Abstract

**Introduction:**

Oral squamous cell carcinoma (OSCC) is a significant global health burden, where timely and accurate diagnosis is essential for improved patient outcomes. Conventional diagnosis relies on manual evaluation of hematoxylin and eosin (H&E)-stained slides, a time-consuming process requiring specialized expertise and prone to variability. While deep learning methods, especially convolutional neural networks (CNNs), have advanced automated analysis of histopathological images for cancerous tissues in various body parts, OSCC presents unique challenges. Its infiltrative growth patterns and poorly defined boundaries, coupled with the complex architecture of the oral cavity, make accurate segmentation particularly difficult. Traditional CNNs which sturggle to capture critical global contextual information often fail to distinguish the complex tissue structures in OSCC images.

**Methods:**

To address these challenges, we propose a novel architecture called **gamUnet**, which integrates the Global Attention Mechanism (GAM) to enhance the model's ability to capture global cross-modal information. This allows the model to focus on key diagnostic regions while retaining detailed spatial information. Additionally, we introduce an extended model, gamResNet, to further improve OSCC detection performance. Both architectures show significant improvements in handling the unique challenges of oral cancer images.

**Results:**

Extensive experiments on public datasets show that our GAM-enhanced architecture significantly outperforms conventional models, achieving superior accuracy, robustness, and efficiency in OSCC diagnosis.

**Discussion:**

Our approach provides an effective tool for clinicians in diagnosing OSCC, reducing diagnostic variability, and ultimately contributing to improved patient care and treatment planning.

## 1 Introduction

Oral squamous cell carcinoma (OSCC) is an aggressive malignancy characterized by high global incidence and mortality (1, 2). OSCC arises from abnormal cellular proliferation within the oral epithelium and represents a significant global health burden, accounting for over 90% of oral malignancies (3–5). Annually, more than 657,000 new OSCC cases are diagnosed, resulting in ~330,000 deaths (6). Timely and accurate diagnosis is critical, as survival rates drastically decrease from 80% in early-stage cases to 20%–30% in advanced stages (7). Currently, OSCC diagnosis primarily relies on microscopic examination of hematoxylin and eosin (H&E)-stained tissue slides (8). However, this manual process is highly labor-intensive, time-consuming and subject to inter-observer variability, which underscores the necessity for automated solutions to improve diagnostic accuracy and consistency.

Deep learning, particularly convolutional neural networks (CNNs), has emerged as a powerful tool for medical image analysis. For instance, U-Net (9) and its variants have achieved notable success in segmenting medical images across modalities such as CT, MRI, and histopathology (10–13). Similarly, ResNet (14) is widely applied for classification tasks, effectively identifying diseases including colorectal cancer, brain tumors, and glioma subtypes (15–17). Building on these successes, CNNs hold promise for OSCC image analysis, potentially enhancing diagnostic efficiency. Previous studies on OSCC segmentation have predominantly utilized variants of U-Net or other CNN-based models, achieving promising results through enhanced architectures, multi-stage approaches, and feature extraction techniques (18–21). For OSCC classification, architectures and transfer learning strategies have been employed (22–27), reporting high accuracy but often facing limited sensitivity, robustness, and generalizability due to small datasets or inconsistent preprocessing. Overall, despite these advances, existing methods for OSCC still encounter significant challenges in effectively balancing accuracy, computational efficiency, and robustness in clinical settings.

The complexity of oral cavity tissue structures introduces unique challenges to OSCC imaging analysis, differentiating it from other cancers characterized by more homogeneous tissues (28). OSCC typically exhibits invasive growth patterns with ill-defined boundaries (29, 30), frequently infiltrating adjacent complex tissues including mucous membranes, bone, and soft tissues (31–33). This invasive behavior complicates tumor boundary delineation, as critical pathological features may span multiple image regions, making it particularly challenging to delineate the exact borders of the tumor. Traditional CNN approaches, like U-Net, ResNet, and exisiting OSCC methods building upon them, relying heavily on localized convolutional operations, often fall short in effectively capturing the global context and long-range dependencies critical for accurately segmenting OSCC. As a result, current automated solutions for OSCC segmentation and classification remain suboptimal

A possible solution is utilizing the attention mechanism. Recently, research has explored incorporating attention mechanisms to enhance CNN performance in image tasks. SENet (34) used channel attention but lacked spatial awareness. CBAM (35) and BAM (36) incorporated spatial attention but couldn't fully capture interactions across all dimensions, and TAM (37) improved this by addressing two dimensions at a time. GAM (38) further advanced this by simultaneously capturing cross-dimensional interactions, making it ideal for handling complex medical images.

However, the application of attention mechanisms, including GAM, to OSCC H&E-stained histopathological image segmentation and classification remains underexplored. Existing research primarily focuses on refining CNN architectures or applying transfer learning without fully addressing the inherent challenges in OSCC imaging, as analyzed aforementioned.

To address these challenges, we propose a novel approach designed specifically for OSCC. We introduce **gamUnet**, an innovative model integrating the Global Attention Mechanism into the U-Net architecture to enhance the capture of global context and cross-dimensional interactions, crucial for delineating complex OSCC tissue structures. Furthermore, we propose **gamResNet**, extending our approach by incorporating residual networks enhanced by GAM to improve OSCC detection. Our proposed models effectively address existing limitations, significantly improving the segmentation and classification accuracy and robustness for OSCC.

Our key contributions are as follows:

- We propose **gamUnet** and **gamResNet**, two novel architectures integrating GAM for improved OSCC segmentation and detection.
- We demonstrate the advantage of global attention mechanism in capturing complex tissue structures and delineating tumor regions, providing insight into the benefits of GAM in medical image analysis.
- Our experimental results validate that our models significantly outperform traditional CNN approaches, offering a promising tool for clinical application in OSCC diagnosis.

## 2 Related work

### 2.1 Deep learning for OSCC analysis

Past studies have examined various deep learning architectures for OSCC segmentation. Martino et al. (18), for instance, introduced the Oral Cancer Annotated (ORCA) dataset and evaluated multiple CNN-based architectures on the dataset, including SegNet, U-Net, and U-Net variants with VGG-16 and ResNet50 encoders, which showed promising results. Dos et al. (19) incorporated color space features into a U-Net-based model for better tumor region identification and background removal, and achieved an impressive accuracy of 97.6% on the Oral Cavity-Derived Cancer(OCDC) dataset. Pennisi et al. (20) designed a Multi-encoder U-Net where input images were divided into tiles and processed by separate encoders before merging, allowing multi-region feature fusion. Musulin et al. (21) proposed a two-stage system for diagnosing OSCC, demonstrating effectiveness in multiclass grading and segmentation. Beyond segmentation, classification of OSCC using deep learning has also seen notable developments. Wang et al. (1) introduced a semi-supervised boundary-aware U-Net with transformation consistency and contrastive learning, outperforming fully-supervised models on the OCDC dataset in low-label regimes. Ünsal et al. (39) applied a U^2^-Net encoder-decoder architecture on private OSCC datasets and reported robust performance with a Dice coefficient of 0.86. Shah et al. (40) proposed OCANet, which incorporates both local and global attention branches to enrich contextual understanding, achieving 86.1% Dice and 77.1% mIoU on ORCA. A range of CNN architectures has been applied to H&E-stained histopathological images for oral cancer detection (22–27), leveraging transfer learning and hybrid models to improve accuracy.

Despite these advancements, OSCC segmentation remains challenging due to the inherent complex anatomical structure of the oral cavity, invasive tumor growth, and ill-defined tumor margins (28–30). While existing OSCC-specific methods have demonstrated promising results, they are often not explicitly designed to address these unique challenges, lacking mechanisms to effectively capture global contextual information, resulting in suboptimal performance. In contrast, our work specifically addresses this challenge utilizing the Global Attention Mechanism.

### 2.2 Attention mechanism in CNNs

In recent years, attention mechanism has garnered significant interest in enhancing CNN-based models to improve the performance of image classification and segmentation tasks, by allowing them to focus on relevant features within an image. Early examples include SENet (34), which pioneered this field by using the channel attention to highlight significant features while suppressing irrelevant ones. However, its primary focus on channel attention restricted its capacity to address spatial information effectively. More recent approaches, such as CBAM (35) and BAM (36), have introduced spatial attention alongside channel attention. Their designed Convolutional Block Attention Module (CBAM) and Bottleneck Attention Module (BAM) have improved model performance in a variety of applications. However, these models still fall short in capturing interactions between channel, spatial width, and spatial height, which are critical for understanding complex medical images. Recognizing the importance of cross-dimension interactions, Misra et al. (37) introduced the triplet attention module (TAM), which considered the relationships between channel, spatial width, and spatial height. Although TAM improved efficiency, it still applied attention to only two dimensions at a time, limiting its ability to fully capture interactions across all three dimensions. Addressing this, Liu et al. (38) introduced the Global Attention Mechanism (GAM) to enhance feature extraction by capturing cross-dimensional interactions across the channel, spatial width, and spatial height simultaneously, making it particularly suited for complex medical imaging tasks that demand comprehensive feature representation. Recently, attention modules have been directly embedded into OSCC segmentation architectures. OCANet (40), for instance, utilizes a dual-branch attention mechanism to integrate local fine-grained detail with global structural awareness.

Still, the potential of attention mechanisms in enhancing OSCC imaging analysis remains underexplored. Many of these models either employ decoupled attention paths or require extensive network modifications, leading to increased computational cost. In contrast, our proposed architecture utilizing GAM offers a lightweight yet expressive approach that integrates seamlessly with CNNs, enabling effective cross-dimensional feature fusion for OSCC segmentation and classification.

### 2.3 Comparative summary of related methods

To provide a clearer understanding of the landscape of OSCC analysis and attention mechanisms in convolutional networks, we summarize representative studies in Table 1. The table categorizes prior works based on dataset, model architecture, core strengths, and limitations. Notably, many OSCC-specific segmentation models leverage U-Net variants, often falling short in capturing comprehensive global context, which is critical for handling the anatomical complexity of OSCC. In parallel, the adoption of advanced attention modules like GAM remains limited in OSCC-focused studies. By proposing a new model architecture integrating GAM, we aim to address this gap and enhance both segmentation accuracy and contextual awareness with minimal architectural overhead.

**Table 1:** Comparison of related OSCC segmentation and attention-based methods.

**Study**	**Dataset**	**Method**	**Strengths**	**Limitations**
Dos et al. (19)	OCDC	U-Net + color space	High accuracy	Dataset-specific tuning
Pennisi et al. (20)	Private, TCGA, ORCA	Multi-encoder U-Net	Multi-region fusion	Limited global semantics
Musulin et al. (21)	Private	Two-stage pipeline	Handles grading and segmentation	Complex pipeline
Wang et al. (1)	OCDC	Semi-supervised U-Net	Label-efficient contrastive learning	Requires transformation consistency
Ünsal et al. (39)	Private	U^2^-Net	High Dice score	No explicit attention mechanism
Shah et al. (40)	ORCA, OCDC, DigestPath	Dual-branch OCANet	Local-global attention fusion	Complex dual-branch structure
SENet (34)	Generic	Channel attention	Lightweight and modular	Ignores spatial features
CBAM (35)	Generic	Channel + spatial attention	Better generalization	Incomplete cross-dimension fusion
BAM (36)	Generic	Channel + spatial attention	Improved feature selection	Lacks full dimension interaction
TAM (37)	Generic	Triplet attention	Captures more dimensions	Still partial fusion
GAM (38)	Generic	Global attention	Full cross-dimension modeling	Not applied to OSCC yet

## 3 Methods

In this study, we introduce a novel model architecture **gamUnet**, and an extended model **gamResNet**, which integrate a Global Attention Mechanism into U-Net and ResNet architectures, respectively. These models specifically target the challenges posed by tumor infiltration and poorly defined lesion boundaries in OSCC histopathological image analysis. Unlike traditional CNN architectures, which excel at capturing local features but fail in modeling global context, the proposed GAM significantly enhances global contextual awareness and captures long-range dependencies.

### 3.1 Base architecture

We adopt U-Net as the base architecture for image segmentation and ResNet-18 for image classification, both of which have demonstrated strong performance in medical image analysis. **U-Net** is selected as the base architecture for segmentation due to its robust performance in medical imaging segmentation. The U-Net architecture employs an encoder-decoder structure, where the encoder progressively reduces the spatial resolution of the input image, capturing abstract features, while the decoder upscales these features to create high-resolution segmentation masks. Skip connections between corresponding layers of the encoder and decoder preserve fine-grained spatial details during the upsampling process. For classification, **ResNet-18** (14) is adopted. Despite having fewer layers compared to deeper ResNet variants, ResNet-18 provides a balanced trade-off between computational efficiency and predictive performance, essential for deployment in clinical settings. ResNet-18, reduces the spatial resolution of the input through convolution and pooling layers, capturing high-level features. A key feature of ResNet-18 is the use of residual (skip) connections, which allow for the efficient training of deeper networks by bypassing certain layers, thereby addressing the vanishing gradient problem. In classification tasks, ResNet-18 outputs class probabilities after reducing the feature map through a fully connected layer.

### 3.2 Global attention mechanism

While both U-Net and ResNet-18 excel in capturing local features, they struggle with modeling global dependencies, a crucial aspect in tasks involving complex medical images in OSCC. To address their limitations in global context modeling, we integrate GAM into both architectures. GAM computes attention across channel and spatial dimensions, significantly enhancing feature representation.

Given a feature map *F*∈*R*^*C*×*H*×*W*^, GAM calculates channel attention *M*_*c*_ and spatial attention *M*_*s*_ as:


(1) $$\begin{array}{lll} M_{c}\left(F\right) & = & \sigma \left(MLP\left(AvgPool\left(F\right)\right)+MLP\left(MaxPool\left(F\right)\right)\right) \end{array}$$

(2) $$\begin{array}{lll} M_{s}\left(F\right) & = & \sigma \left(Conv^{7\times 7}\left(\left[AvgPool\left(F\right);MaxPool\left(F\right)\right]\right)\right) \end{array}$$

Here, σ denotes the sigmoid activation, AvgPool and MaxPool represent global average and max pooling operations, respectively, and *MLP* denotes a multilayer perceptron for channel-wise computations. For an input feature map *F*_1_, GAM computes the intermediate feature map *F*_2_ and the final output *F*_3_ as follows:


(3) $$\begin{array}{lll} F_{2} & = & M_{c}\left(F_{1}\right)⊗F_{1} \end{array}$$

(4) $$\begin{array}{lll} F_{3} & = & M_{s}\left(F_{2}\right)⊗F_{2} \end{array}$$

where ⊗ represents element-wise multiplication.

The channel attention module captures relationships between channels, enhancing important features, while the spatial attention module emphasizes crucial spatial regions. GAM enables the model to focus on regions that are critical for diagnosis, thus enhancing performance, especially in tasks with complex tissue structures like OSCC.

### 3.3 Model variants

We propose two key model variants: **gamUnet** for segmentation tasks and **gamResNet** for classification tasks. These models incorporate the GAM module at different points in their architecture to enhance their ability to capture global context while preserving local feature details.

#### 3.3.1 gamUnet

**Figure 1:**
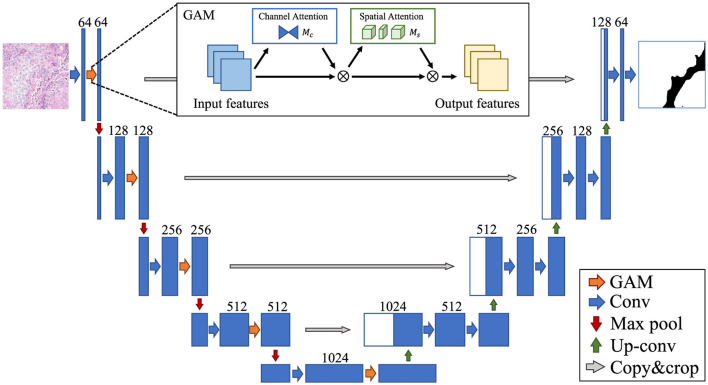
gamUnet.

For segmentation, the GAM module is incorporated into each convolutional block of the U-Net encoder (Figure 1). Each encoder block comprises the following steps: First perform convolution *Conv*^3 × 3^ to obtain preliminary feature maps. Then, apply GAM to these feature maps to compute attention weights. Finally, multiply the attention-weighted features to refine and amplify significant regions. Refined features are passed through skip connections to the decoder, preserving both global context and local spatial details for accurate segmentation.

#### 3.3.2 gamResNet

**Figure 2:**
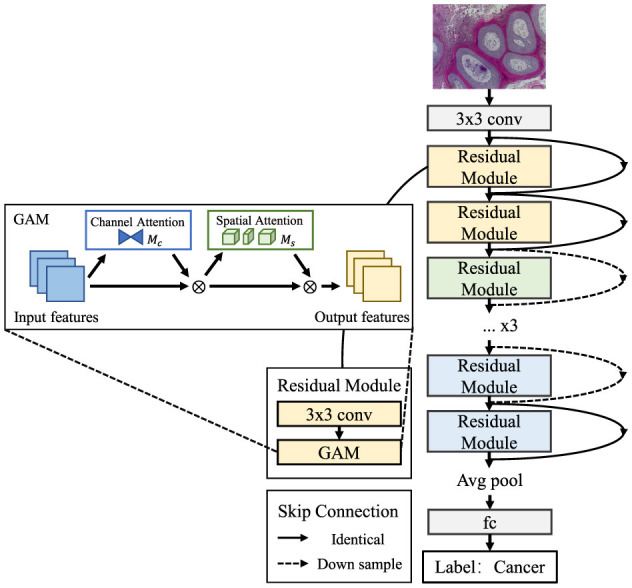
gamResNet.

For gamResNet, GAM modules replace the second convolution within each residual block of ResNet-18 (Figure 2). Specifically: First perform initial convolution *Conv*^3 × 3^ to extract preliminary features. Then replace second convolution with GAM, refining features by computing global attention weights. Finally, utilize the residual connection to combine GAM-enhanced features with the initial block input.

Through these explicit technical refinements, our **gamUnet** and **gamResNet** architectures robustly address the complex and heterogeneous nature of OSCC histopathological images for enhanced segmentation and classification, significantly surpassing traditional CNN methods by effectively capturing critical local and global contextual information.

## 4 Experiments

In this section, we first describe in detail the datasets, training procedures, experimental setup and evaluation metrics, providing a clear explanation of how our models are trained and evaluated, including critical steps involved in data preparation and model training, including image pre-processing, feature extraction, and model optimization. We then present the main experimental results, and the ablation studies conducted to rigorously assess the effectiveness of our proposed gamUnet and gamResNet architectures for OSCC segmentation and classification tasks.

### 4.1 Datasets

#### 4.1.1 Segmentation datasets

**Figure 3:**
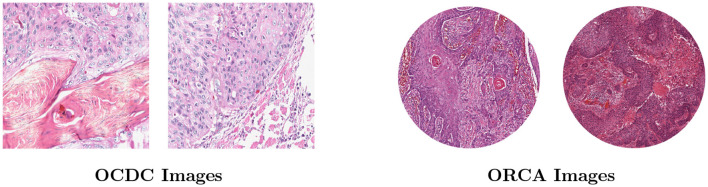
Image examples of OCDC and ORCA datasets.

Table 2 presents the statistics of datasets utilized for segmentation task, Figure 3 presents the image examples.

**Table 2:** Dataset statistics for segmentation.

**Dataset**	**Image size**	**#Train images**	**#Test images**	**Magnification**
OCDC (19)	640 × 640	840 patches	180 patches	20×
ORCA (18)	4,500 × 4,500	100 core images	100 core images	40×

##### 4.1.1.1 OCDC dataset

The OCDC dataset consists of 15 H&E-stained whole slide images (WSIs) derived from human patients diagnosed with OSCC. A total of 1,020 annotated image patches, each measuring 640 × 640 pixels, were extracted from these WSIs, with tumor regions manually marked by a pathologist. This dataset is specifically designed for the automated detection and segmentation of OSCC, featuring a training set of 840 image patches and a test set of 180 patches, all digitized at a 20 × magnification.

##### 4.1.1.2 ORCA dataset

The ORCA dataset includes 200 H&E-stained WSIs sourced from the Cancer Genome Atlas (TCGA) and manually annotated by expert pathologists. Each WSI is composed of one or two cores with a size of 4,500 × 4,500 pixels, containing ground-truth data for tumor pixels. The dataset is split into two subsets: a validation set and a test set, each consisting of 100 core images. In this study, the validation set was utilized for training purposes. Because the original core images only the middle round part is a valid image, so we cut the original image into 1,200*1,200 patches in preprocessing. Then, in order to filter out the low-quality images located at the edges, we removed the patches whose corresponding mask black part of the larger than 80% of the patches, resulting in 728 training images and 796 validation images. The ORCA dataset presents a higher level of complexity due to its larger size and greater complexity, making it suitable for evaluating advanced segmentation models.

#### 4.1.2 Classification datasets

**Figure 4:**
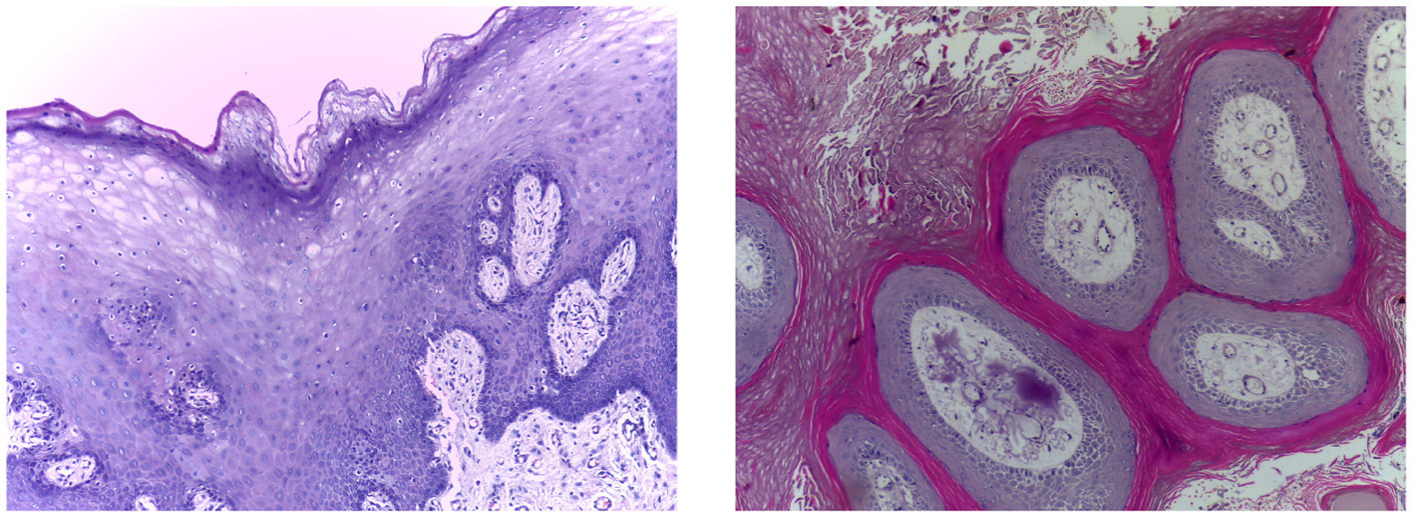
Image examples of the classification dataset. **(Left)** Normal epithelium. **(Right)** OSCC.

Table 3 presents the statistics of the datasets utilized for classification, Figure 4 presents the image examples.

**Table 3:** Dataset statistics for classification.

**Magnification**	**Number of normal images**	**Number of OSCC images**	**Total**
100 ×	89	439	528
400 ×	201	495	696
Total images	290	934	1,224

This dataset (41) comprises a total of 1,224 histopathological images, divided into two distinct sets with varying resolutions. The first set contains 89 images of normal oral epithelium and 439 images of OSCC, all captured at 100 × magnification. The second set includes 201 images of normal oral epithelium and 495 OSCC images, captured at 400 × magnification. All images were acquired using a Leica ICC50 HD microscope from H&E stained tissue slides. These slides were collected, prepared, and cataloged by medical professionals from 230 patients.

### 4.2 Evaluation metrics

We adopt standard evaluation metrics for both segmentation and classification tasks. Accuracy reflects the overall proportion of correctly classified instances and is defined as:


$$\text{Accuracy}=\frac{TP+TN}{TP+TN+FP+FN}$$

Sensitivity (also referred to as recall in classification) quantifies the ability to correctly identify positive samples:


$$\text{Sensitivity/recall}=\frac{TP}{TP+FN}$$

Specificity measures the proportion of actual negatives correctly identified:


$$\text{Specificity}=\frac{TN}{TN+FP}$$

Precision evaluates the proportion of predicted positives that are truly positive:


$$\text{Precision}=\frac{TP}{TP+FP}$$

The F1 score, commonly used in classification, is the harmonic mean of precision and sensitivity:


$$\text{F1 score}=\frac{2\times \text{precision}\times \text{sensitivity}}{\text{precision}+\text{sensitivity}}$$

For segmentation tasks, we also report the Dice coefficient, which shares the same formula as the F1 score:


$$\text{Dice}=\frac{2TP}{2TP+FP+FN}$$

The Jaccard Index or Intersection over Union (IoU) assesses the overlap between predicted and ground truth regions:


$$\text{IoU}=\frac{TP}{TP+FP+FN}$$

For classification, we further report the Area Under the Receiver Operating Characteristic Curve (AUROC), which reflects the model's ability to distinguish between classes by summarizing the trade-off between sensitivity and false positive rate across thresholds.

### 4.3 Baselines

Below are brief descriptions of the baseline algorithms. For image segmentation:

- **U-Net (9)**: an established encoder-decoder CNN architecture widely employed in medical image segmentation, characterized by its symmetrical U-shaped structure.
- **Attention U-Net (42)**: an extension of U-Net that incorporates attention mechanisms to focus on relevant regions and emphasize important features.
- **Depthwise U-Net (43)**: a variant of U-Net that uses depthwise separable convolutions to reduce the number of parameters and computational complexity.
- **Res U-Net (44)**: a U-Net model enhanced with residual connections, which help to alleviate the vanishing gradient problem and improve feature propagation through the network.
- **Shuffle U-Net (45)**: a lightweight U-Net variant that utilizes shuffle operations to improve feature mixing and maintain high segmentation accuracy with reduced computational cost.

For image classification:

- **ResNet (ResNet-18 and ResNet-50) (14)**: residual networks that use skip connections to enable deeper architectures with improved gradient flow.
- **MobileNet-V3-Small (46)**: a compact and efficient CNN model designed for mobile and edge devices, utilizing efficient layers like depthwise convolutions and squeeze-and-excitation blocks.
- **MobileNet-V2 (47)**: a lightweight CNN model featuring inverted residuals and linear bottlenecks, optimized for performance on mobile and low-resource devices.
- **MobileNet-V1 (48)**: the original MobileNet architecture that employs depthwise separable convolutions to reduce model size and complexity.
- **EfficientNet (EfficientNet-B0 and EfficientNet-B1) (49)**: utilizes compound scaling methods that systematically balance depth, width, and resolution, achieving superior accuracy with fewer parameters.

### 4.4 Training and implementation details

**Figure 5:**
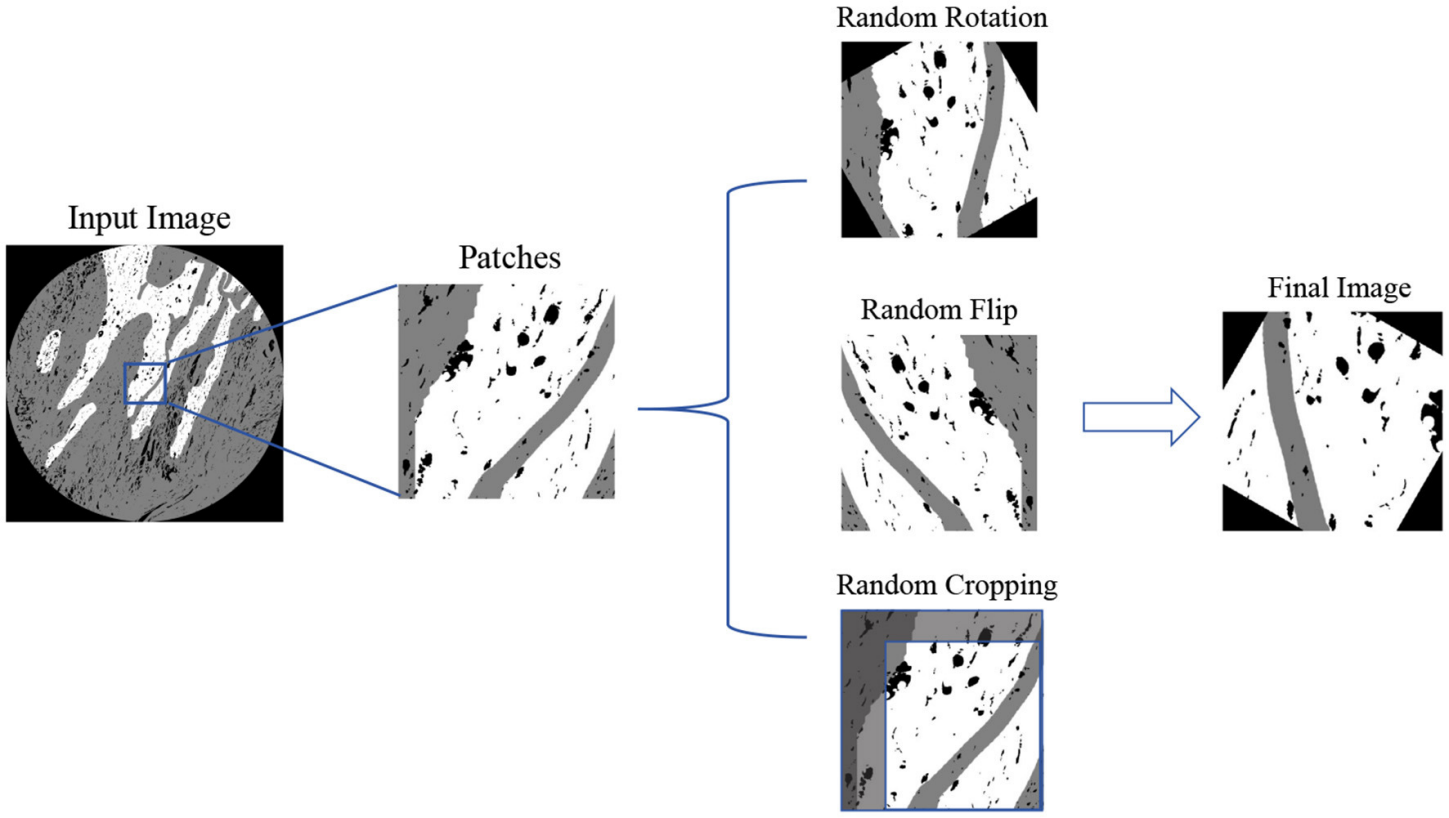
Input image preprocess workflow.

We train all models on both medical image segmentation datasets and medical image classification datasets, including the **OCDC dataset**, the **ORCA dataset** and **100X and 400X classification datasets**. Prior to training, the original images from both datasets were resized to 256 × 256 pixels and augmented using random rotations, flips, and scaling to enhance model robustness. Each image undergoes the process shown in Figure 5.

Models were trained using **binary cross-entropy loss** and optimized using **Adam optimizer**. The training was conducted with a **batch size of 32** and a **learning rate of 0.01**, using **early stopping** to prevent overfitting. Each model's performance was evaluated using key metrics introduced above. All experiments were implemented using PyTorch and conducted on an NVIDIA RTX 3090 GPU with 24GB memory. The classification models were trained for ~5.5 h, while the segmentation models required about 4 h on the OCDC dataset and 1.5 h on the smaller ORCA dataset.

## 5 Results

### 5.1 Experimental results for OSCC segmentation

The segmentation performance on both the OCDC and ORCA datasets, as shown in Tables 4, 5, highlights the effectiveness of our proposed model architecture **gamUnet**, outperforming traditional architectures such as plain **U-Net**, **Attention-Unet**, **Depthwise-Unet**, **Res-Unet**, and **Shuffle-Unet**. Given the class imbalance in the datasets, our primary evaluation metrics are the F1/Dice score and the Intersection-over-Union (IOU, also known as the Jaccard Index), which effectively measure segmentation accuracy under imbalanced scenarios. On the OCDC dataset, **gamUnet** achieves the highest F1/Dice score (0.9096) and IOU (0.8315), indicating enhanced ability to precisely delineate tumor regions while minimizing false predictions. While all models experience a decrease in performance on the more complex ORCA dataset, **gamUnet** maintained robust results with the highest F1/Dice score (0.7047) and IOU (0.5631), demonstrating its resilience against complex anatomical structures and tumor morphology. The superior performance of **gamUnet** highlights its capacity for capturing global context through the integrated GAM, effectively balancing local details with global semantic information crucial for OSCC segmentation tasks.

**Table 4:** Segmentation performance comparison on OCDC dataset.

**Model**	**Acc**	**SE**	**SP**	**PC**	**F1/dice**	**JS/IOU**
Unet (9)	0.9064 (0.0057)	**0.9268 (0.0137)**	**0.9042 (0.0157)**	0.8747 (0.0102)	0.8963 (0.0088)	0.8145(0.0158)
AttentionUnet (42)	0.9126 (0.0058)	0.9117 (0.0147)	0.8965 (0.0157)	0.8682 (0.0102)	0.8906 (0.0088)	0.8042 (0.0158)
DepthwiseUnet (43)	0.8454 (0.0358)	0.8652 (0.0458)	0.8343 (0.0458)	0.8084 (0.0328)	0.8437 (0.0398)	0.7321 (0.0458)
ResUnet (44)	0.9173 (0.0168)	0.9191 (0.0258)	0.9015 (0.0288)	0.8747 (0.0238)	0.9024 (0.0198)	0.8253 (0.0208)
ShuffleUnet (45)	0.8565 (0.0568)	0.9027 (0.0358)	0.8593 (0.0538)	0.8374 (0.0328)	0.8445 (0.0298)	0.7563 (0.0328)
gamUnet	**0.9184 (0.0058)**	0.9216 (0.0147)	0.9035 (0.0157)	**0.8847 (0.0102)**	**0.9096 (0.0088)**	**0.8315 (0.0158)**

The best results are highlighted in bold. The reported values represent the average of four runs, with the standard deviation shown in parentheses.

**Table 5:** Segmentation performance comparison on OCRA dataset.

**Method**	**Acc**	**SE**	**SP**	**PC**	**F1/Dice**	**JS/IOU**
Unet (9)	0.7446 (0.0458)	0.7750 (0.0257)	0.7064 (0.0988)	**0.6522 (0.0608)**	0.6821 (0.0508)	0.5362 (0.0508)
AttentionUnet (42)	0.7325 (0.0428)	0.7749 (0.0298)	0.6712 (0.0628)	0.6342 (0.0528)	0.6683 (0.0508)	0.5271 (0.0428)
DepthwiseUnet (43)	0.7038(0.0408)	0.7865 (0.0388)	0.6309(0.0538)	0.6018 (0.0238)	0.6635 (0.0398)	0.5226 (0.0458)
ResUnet (44)	**0.7847 (0.0088)**	0.7857 (0.0458)	**0.7452 (0.0288)**	0.6115 (0.0198)	0.6929 (0.0208)	0.5478(0.0138)
ShuffleUnet (45)	0.7275 (0.0168)	0.7508 (0.0458)	0.6607 (0.0388)	0.6042(0.0328)	0.6683 (0.0298)	0.5162 (0.0208)
gamUnet	0.7496 (0.0228)	**0.8196 (0.0458)**	0.6712 (0.0408)	0.6218 (0.0328)	**0.7047 (0.0398)**	0.5631 (0.0328)

The best results are highlighted in bold. The reported values represent the average of four runs, with the standard deviation shown in parentheses.

**Figure 6:**
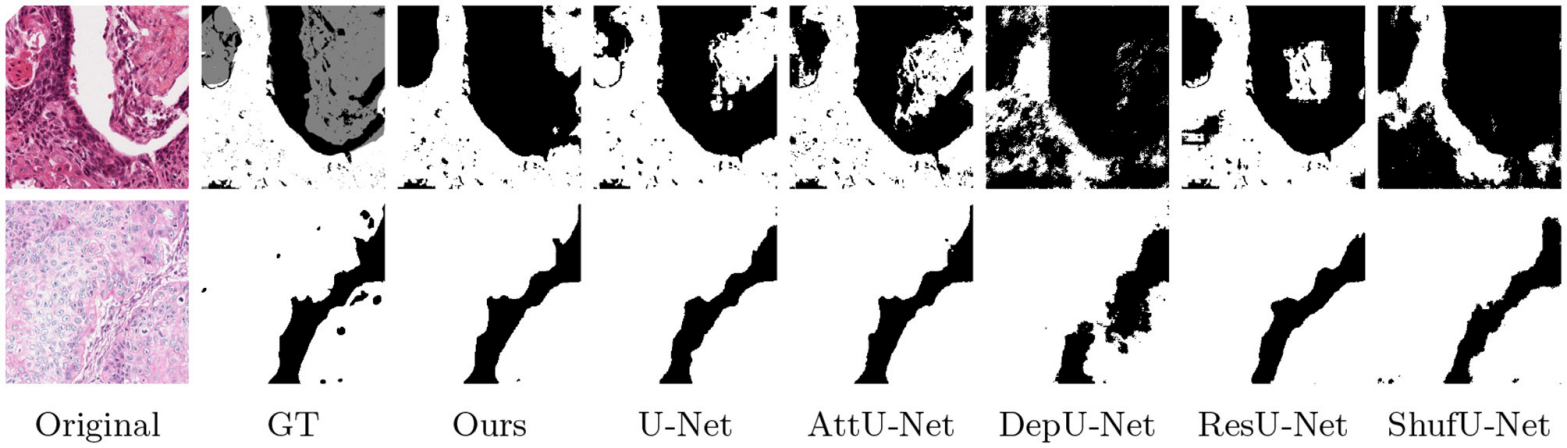
Segmentation performance evaluation of various models for oral cancer detection using the OCDC dataset. The classes are defined as: white for carcinoma pixels and black for non-carcinoma pixels. The bar chart displays the average performance of each model on the test set.

Figure 6 presents qualitative and visualized segmentation results on the OCDC dataset. Compared to baselines, **gamUnet** yields segmentation masks that more closely align with the ground truth, effectively capturing tumor boundaries. In contrast, other models exhibit either over-segmentation or structural inaccuracies, particularly in complex regions. These results further validate the advantage of incorporating global attention for precise and robust OSCC segmentation.

### 5.2 Experimental results for OSCC classification

Tables 6, 7 detail the classification performance evaluated at 100X and 400X magnifications on the oral cancer classification datasets. Considering the dataset imbalance, AUROC and F1 Score are the key evaluation metrics. On the 100X dataset, our proposed **gamResNet** attained the highest AUROC (0.991), accuracy (0.902), and F1 Score (0.945), outperforming traditional baselines such as ResNet-50, MobileNet-V2, and EfficientNet-B1. This underscores **gamResNet**'s superior capacity to accurately distinguish between positive and negative samples. Additionally, mobilenet-v1 and efficientnet-b0 exhibit competitive F1 scores (0.897 and 0.898, respectively), although they fall behind in terms of AUROC and accuracy, indicating a trade-off in model performance across different metrics.

**Table 6:** Classification performance comparison on OCDC 100X dataset.

**Model**	**AUROC**	**Accuracy**	**Precision**	**Sensitivity**	**Specificity**	**F1 Score**
ResNet-50 (14)	0.827 (0.045)	0.818 (0.049)	0.818 (0.032)	0.993 (0.006)	0.215 (0.215)	0.900 (0.026)
ResNet-18 (14)	0.924 (0.031)	0.873 (0.029)	0.913 (0.008)	0.933 (0.027)	0.614 (0.331)	0.923 (0.032)
MobileNet-v3-small (46)	0.731 (0.032)	0.800 (0.056)	0.815 (0.047)	0.978 (0.015)	0.232 (0.154)	0.889 (0.027)
MobileNet-v2 (47)	0.902 (0.037)	0.855 (0.072)	0.951 (0.014)	0.867 (0.037)	**0.800 (0.165)**	0.907 (0.038)
MobileNet-v1 (48)	0.853 (0.045)	0.836 (0.067)	**0.929 (0.022)**	0.867 (0.043)	0.712 (0.576)	0.897 (0.056)
EfficientNet-b1 (49)	0.724 (0.088)	0.818 (0.036)	0.818 (0.039)	0.989 (0.010)	0.345 (0.207)	0.900 (0.047)
EfficientNet-b0 (49)	0.840 (0.054)	0.818 (0.067)	0.830 (0.041)	0.978 (0.007)	0.206 (0.157)	0.898 (0.065)
gamResNet	**0.991(0.015)**	**0.902 (0.022)**	0.896 (0.047)	**0.995 (0.005)**	0.375 (0.456)	**0.945 (0.044)**

The best results are highlighted in bold. The reported values represent the average of four runs, with the standard deviation shown in parentheses.

**Table 7:** Classification performance comparison on OCDC 400X dataset.

**Model**	**AUROC**	**Accuracy**	**Precision**	**Sensitivity**	**Specificity**	**F1 score**
Resnet-50 (14)	0.860 (0.056)	0.800 (0.065)	**0.905 (0.037)**	0.844 (0.018)	0.600 (0.324)	0.874 (0.030)
Resnet-18 (14)	0.848 (0.043)	0.831 (0.032)	0.852 (0.052)	0.920 (0.038)	0.619 (0.420)	0.885 (0.049)
MobileNet-v3-small (46)	0.780 (0.054)	0.775 (0.067)	0.827 (0.076)	0.860 (0.027)	0.571 (0.154)	0.843 (0.036)
MobileNet-v2 (47)	0.711 (0.042)	0.634 (0.084)	0.722 (0.132)	0.780 (0.068)	0.286 (0.165)	0.750 (0.049)
MobileNet-v1 (48)	0.627 (0.053)	0.704 (0.090)	0.704 (0.144)	0.988 (0.011)	0.203 (0.154)	0.826 (0.065)
EfficientNet-b1 (49)	0.724 (0.084)	0.831 (0.053)	0.852 (0.069)	0.979 (0.010)	0.286 (0.202)	0.892 (0.053)
EfficientNet-b0 (49)	0.875 (0.077)	0.848 (0.089)	0.827 (0.087)	0.957 (0.023)	0.600 (0.246)	0.863 (0.071)
gamResNet	**0.924(0.026)**	**0.899 (0.035)**	0.875 (0.043)	**0.991 (0.006)**	**0.650 (0.342)**	**0.933 (0.052)**

The best results are highlighted in bold.

On the 400X dataset, similar trends are observed. GamResNet again achieves the highest AUROC (0.924), accuracy (0.899), and F1 score (0.933), coupled with notably high sensitivity (0.991), which is essential for clinical cancer detection applications. Although efficientnet-b0 performs well with an AUROC of 0.875 and an F1 score of 0.863, it is still notably outperformed by gamResNet. The baseline ResNet-50 model achieves the highest precision (0.905) on the 400X dataset, indicating its capability to minimize false positives, but it falls behind in sensitivity and F1 score.

Collectively, these results demonstrate that integrating the GAM mechanism significantly enhances model robustness and generalization across varied magnification levels and dataset complexities, confirming the clinical utility of **gamResNet** for reliable OSCC classification.

### 5.3 Ablation studies

To investigate the contribution of the GAM in our architectures, we conducted ablation studies on both **gamUnet** and **gamResNet**.

**For gamUnet**, we selectively integrated the GAM modules into the encoder, decoder, or both components of the U-Net architecture. All experiments were conducted under identical training settings on the OCDC dataset. As shown in Table 8, the variant with GAM integrated solely in the encoder (**gamUnet**) achieved the best performance across key segmentation metrics, including accuracy (0.9294), F1/Dice score (0.9168), and Jaccard Index (0.8467). These results highlight the benefit of enhancing global contextual representation at the encoding stage. Conversely, applying GAM to both encoder and decoder improved specificity, suggesting enhanced ability in distinguishing background regions, albeit with a slight drop in overall accuracy. These findings support our design choice of encoder-focused GAM integration.

**Table 8:** Segmentation performance comparison of gamUnet variants on OCDC.

**Model**	**Acc**	**SE**	**SP**	**PC**	**F1/dice**	**JS/IOU**
Unet	0.9101	0.9421	0.8853	0.8571	0.8974	0.8144
Unet + decoder GAM	0.9161	0.9183	0.9095	0.8849	0.9012	0.8201
Unet + encoder and decoder GAM	0.9208	0.9049	**0.9312**	**0.9035**	0.9037	0.8248
gamUnet	**0.9294**	**0.9447**	0.9176	0.8906	**0.9168**	**0.8467**

The best results are highlighted in bold.

**For gamResNet**, we explored how different placements of the GAM module within the residual blocks affect classification performance. The original design of gamResNet replaces the second convolution layer of each residual block with a GAM module. We compared this configuration against two variants: (1) replacing the first convolution instead of the second, and (2) replacing both convolutions with GAM. Results on OCDC 100X dataset are summarized in Table 9. The original gamResNet achieved the best trade-off across all metrics, with the highest AUROC, accuracy, and F1 score, while maintaining a balanced sensitivity and specificity. Replacing only the first convolution slightly reduced AUROC (0.895), indicating suboptimal background discrimination. Substituting both convolutions with GAM led to near-perfect sensitivity but drastically reduced specificity (0.125), suggesting severe over-sensitivity to positive class predictions. These results confirm the efficacy of selectively applying GAM to the second convolutional layer, balancing attention to relevant features without compromising classification reliability.

**Table 9:** Classification performance comparison of gamResNet variants on the OCDC 100X dataset.

**Model variant**	**AUROC**	**Accuracy**	**Precision**	**Sensitivity**	**Specificity**	**F1 Score**
First Conv → GAM	0.895	0.882	0.894	0.977	0.375	0.933
Both Convs → GAM	0.922	0.863	0.860	0.998	0.125	0.925
**gamResNet**	**0.991**	**0.902**	**0.896**	0.995	**0.375**	**0.945**

Best results are highlighted in bold.

Collectively, our ablation studies across segmentation and classification tasks demonstrate that a targeted integration of GAM as designed in our **gamUnet** and **gamResNet** strikes the best balance between leveraging global attention and preserving task-specific structural inductive biases.

### 5.4 Statistical significance

To evaluate the statistical significance and robustness of our experimental results, we conducted four independent runs for each model configuration and report both the mean and standard deviation in the main results. The relatively small standard deviations across most metrics indicate high stability and reproducibility of our results, which also enhances tthe credibility of the comparisons. Additionally, for the OCDC segmentation task, we performed statistical analysis by calculating 95% confidence intervals and *p*-values for key metrics, including F1/Dice and IOU. As summarized in Table 10, our proposed model **gamUnet** achieves significantly higher F1/Dice and IOU scores with narrow confidence intervals and *p*-values well below 0.05. These findings confirm that the observed performance gains are not only consistent but also statistically significant, reinforcing the effectiveness and reliability of our approach in handling complex and imbalanced OSCC image analysis tasks.

**Table 10:** Statistical significance analysis on OCDC segmentation results.

**Model**	**Metric**	**95% CI (low–high)**	***p*-value**
Unet	F1/dice	**[0.8907, 0.9108]**	5.72E-07
	IOU	[0.8033, 0.8365]	3.42E-06
AttentionUnet	F1/dice	[0.8680, 0.8924]	1.11E-06
	IOU	[0.7699, 0.8085]	6.47E-06
DepthwiseUnet	F1/dice	[0.7365, 0.8942]	0.000418
	IOU	[0.6030, 0.8119]	0.001412
ResUnet	F1/dice	[0.8644, 0.9016]	5.08E-06
	IOU	[0.7665, 0.8233]	2.48E-05
ShuffleUnet	F1/dice	[0.7862, 0.9028]	0.000148
	IOU	[0.6576, 0.8246]	0.000629
**gamUnet**	F1/dice	[0.8826, 0.9067]	7.375E-05
	IOU	**[0.7950, 0.8319]**	0.000205

Each value corresponds to the 95% confidence interval and *p*-value for key evaluation metrics. These values were bolded to highlight results that meet the significance threshold (*p* < 0.05).

### 5.5 Model efficiency and computational cost

Regarding the computational overhead introduced by the GAM module, we provide a detailed comparison of model complexity in terms of parameter count across both classification and segmentation tasks.

For segmentation, Table 11 shows that our proposed **gamUnet** contains around 28.9 million parameters. Although this is higher than vanilla Unet (6.8M) and lightweight variants such as Shuffle Unet (0.61M), it remains substantially more efficient than heavy models that similarly incorporates attention mechanisms like Attention Unet (34.9M), while achieving better performance.

**Table 11:** Parameter counts for segmentation models.

**Model**	**Parameters (millions)**
gamUnet	28.95
Unet	6.82
Attention Unet	34.88
ResUnet	1.38
Depthwise Unet	0.61
Shuffle Unet	0.61

For classification, Table 12 shows that our classification model **gamResNet** contains ~8.4 million parameters, significantly fewer than standard ResNet-18 (11.2M) and ResNet-50 (25M), while still outperforming them. Compared to lightweight models such as MobileNet or EfficientNet-B0, **gamResNet** maintains a reasonable parameter size and achieves superior diagnostic accuracy, striking a strong balance between efficiency and effectiveness.

**Table 12:** Parameter counts for classification models.

**Model**	**Parameters (millions)**
gamResNet	8.39
ResNet-18	11.17
ResNet-50	25.00
MobileNet-V1	4.20
MobileNet-V2	3.50
MobileNet-V3-Small	2.54
EfficientNet-B0	5.30
EfficientNet-B1	7.80

Importantly, our design selectively integrates GAM in a minimal yet effective way—encoder-only for **gamUnet** and second-conv-only for **gamResNet**—thereby avoiding unnecessary overhead while preserving the benefits of global attention. This makes our models well-suited for clinical deployment, where both accuracy and resource efficiency are critical.

## 6 Discussion

The experimental results demonstrate that our gamUnet and gamResNet consistently outperformed baselines across key metrics in both segmentation and classification of OSCC histopathological images. The ablation studies also confirms the effectiveness of our designed model architecture. In classification, the gamResNet consistently achieved the highest AUROC and F1 scores across both magnifications. Notably, the enhanced model's ability to maintain high sensitivity across different magnifications underscores its versatility in handling datasets with varying levels of detail, which is highly important given the variability in histopathological images, where different magnification levels can emphasize different cellular structures. By incorporating GAM, our models effectively address the inherent limitations of conventional CNNs, which struggle with capturing global context and long-range dependencies. This enhancement is particularly crucial for tasks on OSCC histopathological images, where tumors could be ill-defined and essential diagnostic features may span across multiple regions of an image.

Overall, our results validate the effectiveness of the proposed model architecture in enhancing the diagnostic accuracy and reliability of deep learning models in OSCC analysis. This approach not only offers a more efficient alternative to manual examination but also has the potential to streamline diagnostic workflows, supporting pathologists in making timely and informed decisions. Future work may involve exploring the application of GAM in other cancer types and expanding the dataset to further validate model robustness. Additionally, integrating multi-scale attention mechanisms could further refine the model's ability to adapt to different magnifications and improve clinical applicability.

## 7 Conclusion

This study presents a deep learning-based approach to enhance the accuracy and efficiency of OSCC diagnosis and segmentation using histopathological images. By designing novel model architectures gamUnet and gamResnet that integrate Global Attention Mechanism to enable the model to capture global information, our method significantly improves the segmentation and classification performance on OSCC H&E-stained images, where ill-defined tumors and cell infiltration pose great challenges for other models. Experimental results on three datasets demonstrate that our gamUnet and gamResNet outperform traditional architectures, effectively distinguishing tumor regions within the complex tissue structures characteristic of OSCC. The ability of our method to provide reliable and consistent results highlights their potential as a diagnostic tool, supporting pathologists in delivering timely and accurate assessments. This work contributes to the advancement of automated pathology tools, aiming to improve patient outcomes in oral cancer care and can be further expanded to other cancer types and more diverse datasets.

## Data Availability

The original contributions presented in the study are included in the article/supplementary material, further inquiries can be directed to the corresponding author.
